# Optimization of the Microwave Assisted Glycosylamines Synthesis Based on a Statistical Design of Experiments Approach

**DOI:** 10.3390/molecules25215121

**Published:** 2020-11-04

**Authors:** Jo Sing Julia Tang, Kristin Schade, Lucas Tepper, Sany Chea, Gregor Ziegler, Ruben R. Rosencrantz

**Affiliations:** 1 Fraunhofer Institute for Applied Polymer Research IAP, Biofunctionalized Materials and (Glyco) Biotechnology, Geiselbergstr. 69, 14476 Potsdam, Germany; josing.tang@iap.fraunhofer.de (J.S.J.T.); kristin.schade@iap.fraunhofer.de (K.S.); sany.chea@iap.fraunhofer.de (S.C.); gregor.ziegler@iap-extern.fraunhofer.de (G.Z.); 2 Department of Physics, Freie Universität Berlin, Arnimallee 14, 14195 Berlin, Germany; tepperlucas0@gmail.com

**Keywords:** carbohydrates, glycosides, glycosylamines, design of experiments (DoE), microwave chemistry, amination

## Abstract

Glycans carry a vast range of functions in nature. Utilizing their properties and functions in form of polymers, coatings or glycan derivatives for various applications makes the synthesis of modified glycans crucial. Since amines are easy to modify for subsequent reactions, we investigated regioselective amination conditions of different saccharides. Amination reactions were performed according to Kochetkov and Likhoshertov and accelerated by microwave irradiation. We optimized the synthesis of glycosylamines for *N*-acetyl-d-galactosamine, d-lactose, d-glucuronic acid and l-(−)-fucose using the design of experiments (DoE) approach. DoE enables efficient optimization with limited number of experimental data. A DoE software generated a set of experiments where reaction temperature, concentration of carbohydrate, nature of aminating agent and solvent were investigated. We found that the synthesis of glycosylamines significantly depends on the nature of the carbohydrate and on the reaction temperature. There is strong indication that high temperatures are favored for the amination reaction.

## 1. Introduction

Glycosylation is a crucial modification of biomolecules involved in almost all biological processes [1,2,3,4,5]. Glycans may act as scaffolds for mechanical stabilization, as cell-surface coating, enabling cellular crosstalk and have various functions including in diseases [6,7,8,9,10,11]. Especially for the latter, potent inhibitors of glycan-binding proteins (lectins) are sought after as well as glycan scaffolds for trapping pathogens [12,13,14]. For all examples, the glycans may be chemically modified and presented in polymers [15,16,17], on surfaces [18,19,20,21,22], on nanoparticles [23,24,25] or as (multivalent) glycan derivatives [26,27,28,29] with increased binding affinity [30,31,32]. Prerequisites for this are straight forward chemical processes that yield regioselective modifications of glycans without hampering the natural recognition processes. For this, very diverse chemical routes have been employed which can be roughly distinguished between protecting group dependent and protecting group free or even enzymatic routes [33,34,35,36]. Protecting group free routes in general require less synthesis steps, but the reaction conditions and purification must be elaborated carefully. However, we utilized a protecting group free process to regioselectively insert an amino group into saccharides at the C1-position which was subsequently modified into a methacrylamide to generate glycopolymers [37,38]. From literature and our work, amination seems a rather robust process, but it turned out that chosen reaction conditions influence the yield substantially. Interestingly, this effect was diverse for different carbohydrates. This amination was introduced by Kochetkov and later modified by Likhoshertov [39,40,41,42]. The Kochetkov reaction is performed with ammonium carbonate whereas the amination according to Likhoshertov employs ammonium carbamate as the aminating agent. Significant advantages of these methods are enabling of protecting group free synthesis routes, the regioselectivity and the applicability on various oligosaccharides with only few and cost-efficient reagents. Essentially, a saccharide is stirred in solvent with an excess amount of amination agent. It is a straight forward approach to regioselectively insert a single functional group into various glycans and enables subsequent coupling to generate glycoconjugates. The Kochetkov amination is further facilitated by employing the advantageous features of microwave assisted synthesis. The reaction can be tremendously accelerated by microwave irradiation, shortening the initial reaction time of 5 d to 90 min [18,43]. Moreover, the use of microwave irradiation allows the tenfold reduction of the amount of ammonium salt, facilitating homogeneous suspending of starting material and purification [43]. To the best of our knowledge, the amination according to Likhoshertov has not been performed under microwave irradiation yet. Here, we investigate this synthesis using microwave irradiation as well. As the syntheses have a broad substrate scope and are only a one-step procedure, they seem a very worthwhile approach to yield glycan derivatives for follow-up functionalization to achieve glycomonomers, biosensor coatings and others. We chose a statistical approach to efficiently determine the optimal amination conditions of saccharides and to study the use of design of experiment (DoE) for optimization of glycochemistry reactions.

Design of experiments is a valuable tool to limit the amount of data needed to find optimal experimental conditions. Any method to optimize a synthesis of interest starts by identifying the parameters of the reaction, namely, temperature, concentration or reaction time. In a classical optimization setting, all but one parameter are kept constant at a time, and the result of the experiment, such as yield or purity, is improved. This strategy, referred to as “one-variable-at-a-time” (OVAT), can be unnecessarily labor-intensive and fails to capture correlations between the input parameters. If these input factors influence each other strongly, OVAT might not find the true optimum of the experimental conditions and the result depends on the initial reaction conditions selected [44]. To circumvent this obstacle, we use a statistical design of experiments approach as an alternative to the OVAT method. DoE aims to evenly sample all possible values for the input parameters and find a mathematical relationship between them and the outcome of the experiment. Although it has been known since the early 1900s, it has only recently found wide-spread application [45,46,47,48,49,50,51]. DoE was previously employed to optimize synthetic procedures with a small number of experiments [50,51,52,53,54,55]. A successful application of DoE guides the selection of further experiments and allows the localization of most promising sets of features. It has become increasingly accessible to researchers through the advent of user-friendly software options such as MODDE or JMP.

Contrary to former studies, where amination was mostly optimized for one specific carbohydrate [18,43,56], we show the significance of and possible interactions between selected parameters for each respective saccharide as the yield and optimal reaction conditions are strongly determined by the nature of chosen saccharide [18,40,43,56,57,58]. For instance, Likhoshertov et al. yielded 81% aminated d-glucuronic acid, while the amination of l-fucose resulted in a yield of 52% with the same reaction conditions [40]. By utilizing the DoE software MODDE, we optimized the reaction conditions for four selected saccharides: *N*-acetyl-d-galactosamine (GalNAc), d-lactose (Lac), d-glucuronic acid (GlcA) and l-(−)fucose (Fuc). These saccharides are important for biomolecular interactions on the one hand and, on the other, they resemble an overview of the most common chemical properties of non-modified glycans such as *N*-acetyl glycans, disaccharides, uronic acids and desoxy-glycans.

## 2. Results and Discussion

### 2.1. Optimizing the Amination of Oligosaccharides

**Scheme 1:**
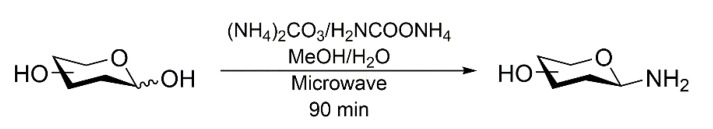
Protecting group free and microwave-assisted synthesis route for amination of free saccharides according to Kochetkov and Likhoshertov in methanol or water with a 5-fold excess of ammonium salt.

We optimized the synthesis of glycosylamines using a statistical DoE approach. As our synthesis route, we chose the amination methods of Kochetkov and Likhoshertov assisted by microwave irradiation (Scheme 1).

To promote an equal distribution of microwave irradiation for all experiments, the volume of solvent was kept constant. We chose to vary reaction temperature, concentration of starting material, solvent and ammonium salt as our quantitative and qualitative parameters (Table 1). Ranges of temperature and concentration were set to 30–60 °C and 10–100 mg/mL, respectively, as the conditions of previous studies mostly lie within these ranges. Former studies showed successful amination of saccharides in water, dimethyl sulfoxide and methanol [18,39,43,56,58,59,60,61]. We tested water and methanol as solvent, since they are more readily removed by evaporation than dimethyl sulfoxide. In addition, ammonium salts and unmodified oligosaccharides generally dissolve better in water than in organic solvents, which might be beneficial for reaction and yield. The other qualitative parameters are the aminating agents ammonium carbonate and ammonium carbamate.

**Figure 1:**
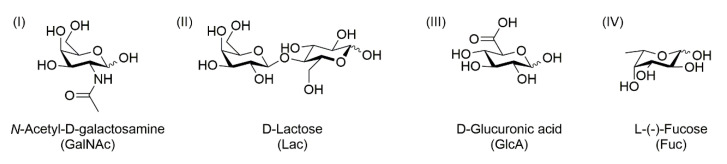
Mono- and disaccharides chosen for the optimization of amination reactions.

We tested the optimization conditions on four chosen saccharides: (I) *N*-acetyl-d-galactosamine (GalNAc), (II) d-lactose (Lac), (III) d-glucuronic acid (GlcA) and (IV) l-(−)-fucose (Fuc) (Figure 1).

The products were not isolated but solvents were fully and ammonium salts were partially or mostly removed under high vacuum. We determined the yields by ^1^H-NMR spectroscopy in deuterium oxide. Here, the peak of the anomeric proton of glycosylamine was analyzed in relation to a known peak that both starting material and glycosylamine share, for example, the methyl moiety of GalNAc/GalNAcNH_2_. NMR spectroscopy offers fast and easy analysis without requiring the isolation of products and is sufficient for the optimization process. However, it is known that glycosylamines can hydrolyze in D_2_O which could distort the actual yield. The hydrolysis rate is decreased with higher pH value [59]. Experiments performed with high amounts of aminating agents can lead to residuals of them after drying and therefore to higher pH values. Due to the basic conditions, less hydrolysis might occur which does not distort the yield as much as experiments performed with low amounts of ammonium salts.

We used the DoE software MODDE to design a set of experiments with varied reaction parameters for optimization. MODDE provides a summary of fit with four values which estimate how well the respective model works. R^2^ indicates how well the model fits the data and should be of large value for a good model. An R^2^ of 0.5 presents a model with rather low significance. The prediction value Q^2^ estimates the predictive power of the model and is the most sensitive indication. Here, a value above 0.1 represents a significant model whereas a value above 0.5 expresses a good model. However, Q^2^ should not deviate from R^2^ by more than 0.3. A model validity of 1.0 represents a perfect model. If the model validity is below 0.25, there are indications of statistically significant problems with the model. Values above 0.25 show that the model error is in the same range as the pure experimental error. The reproducibility value represents the experimental error according to the deviation of responses of repeated experiments and should be above 0.25. MODDE displays a coefficient plot where the significance of chosen factors and their interactions is shown (Supporting Information). We removed non-significant terms from the model.

### 2.2. Design of Experiment Approach

**Figure 2:**
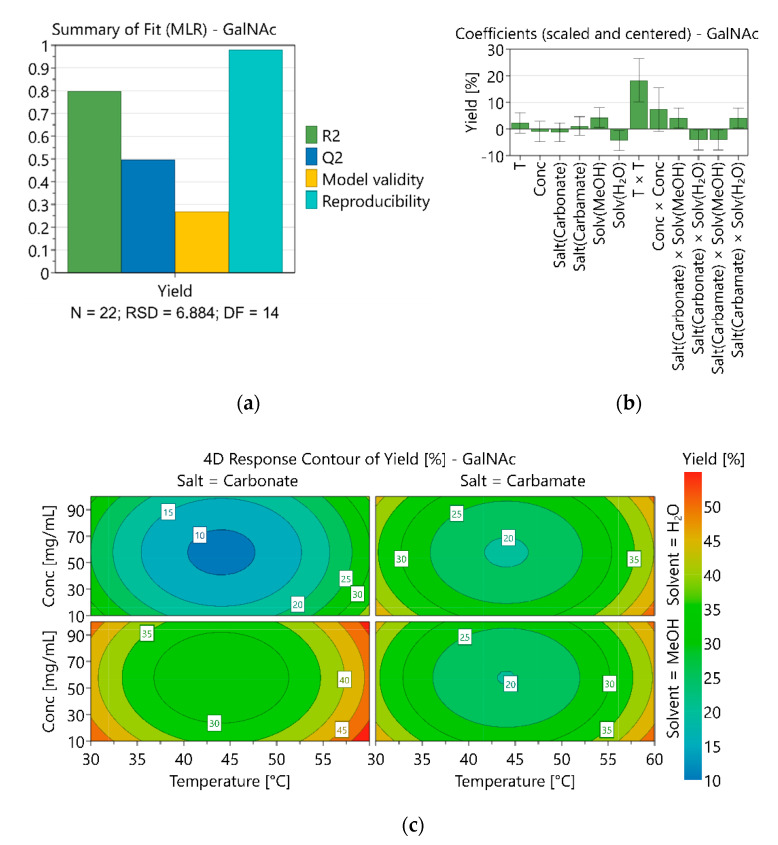
Plots of the model for GalNAc generated by MODDE: (**a**) Summary of fit shows a rather low significance of the model; (**b**) plot of coefficient values for scaled and centered factors shows significant factors according to the model; (**c**) the 4D response contour plot of yield predicts yields of amination in dependence on qualitative and quantitative factors.

The amination of GalNAc was investigated as this saccharide is not only a model compound for 2-*N*-acetylated sugars, but also an important saccharide in mucin-like *O*-glycosylation. In the experimental set for GalNAc, we recognized the data of the experiments Am-I-06 and Am-I-10 (Table 1) as outliers and removed them from the model. The summary of fits of GalNAc (Figure 2a) presents an R^2^ value of 0.80 and a Q^2^ value of 0.50, which indicates a good model. The model validity of 0.27 is rather low; the reproducibility displays a very good value of 0.98. The model validity might be low due to the great reproducibility value. Overall, this model of GalNAc is significant. Significant terms according to MODDE are temperature, concentration, both aminating agents ammonium carbonate and carbamate, the solvents methanol and water and the quadratic term of temperature × temperature, concentration × concentration, ammonium carbonate × water, ammonium carbamate × methanol and ammonium carbamate × water (Figure 2b). The 4D contour plot represents predicted response values as a function of chosen (and significant) factors. Figure 2c shows the yield as a function of concentration (Y-axis) and temperature (X-axis) for both ammonium salts and both solvents, respectively. According to this, temperature and concentration greatly influence the yield. Ammonium carbonate affects the yield only when different solvents are compared. Amination with ammonium carbamate is similar in both water and methanol. We found that the highest yield (64.2%) is achieved at the highest chosen temperature (60 °C) and at the lowest tested concentration (10 mg/mL) with ammonium carbonate and methanol. MODDE calculated optimized conditions with exactly the same reaction conditions and a predicted yield of 54.7%. The predicted yield differs from the achieved one by more than the error deviation; additionally, the calculated optimized yield is lower than the highest yield achieved. This indicates statistical problems of this model. Considering the quantity of varied parameters, a rather small set of experiments has been conducted. A larger number of experiments can improve the model. Since the experimental conditions with methanol and ammonium carbonate proved to be superior, we suggest the collecting of additional data for mentioned condition to further improve the model and optimize the amination conditions for GalNAc.

**Figure 3:**
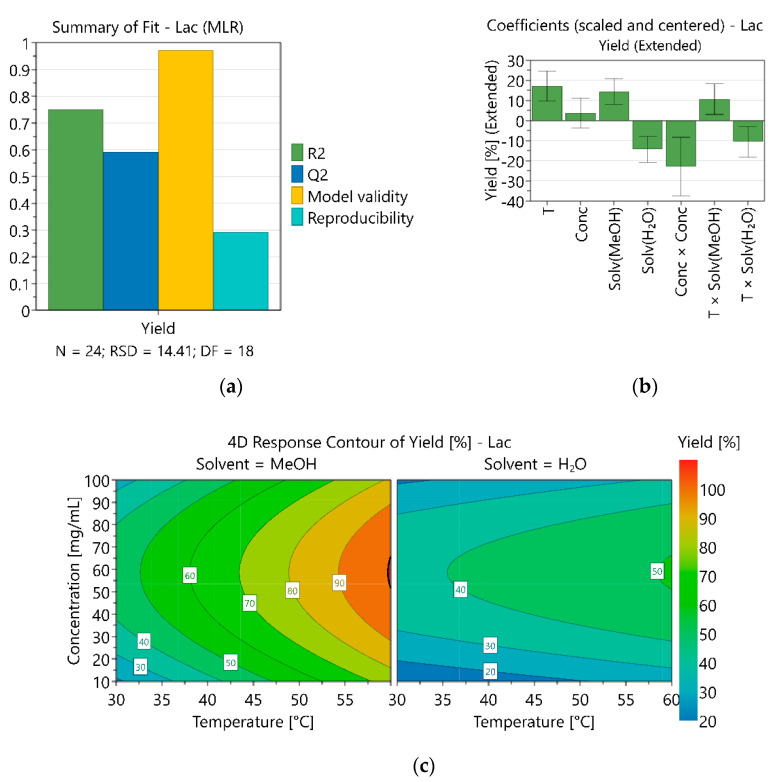
Plots of the model for Lac generated by MODDE: (**a**) Summary of fit represents a good model; (**b**) plot of coefficient values for scaled and centered factors shows significant factors according to the model; (**c**) the 4D response contour plot of yield predicts yields of amination in dependence on qualitative and quantitative factors.

Next, we investigated the reaction of Lac, which is our model compound for disaccharides and also an important ligand for lectins, mostly due to the terminal Gal residue. The summary of fits of the model for Lac shows good values with R^2^ = 0.75 and Q^2^ = 0.59 (Figure 3a). It has an excellent model validity of 0.97 and a low reproducibility of 0.29. Thus, we understand the model for Lac has high significance. Significant terms are temperature, concentration, the solvents methanol and water, the quadratic term of concentration × concentration, temperature × methanol and temperature × water (Figure 3b). Hence, the amination of Lac is less dependent on the nature of ammonium salt than on the other factors. The 4D response contour plot for Lac shows that the yield increases with rising temperature and with a concentration converging at 58.3 mg/mL (Figure 3c). We can clearly observe a strong dependence of the yield on temperature and less on concentration. Furthermore, the plot indicates that temperatures above 60 °C may lead to even better yields. Surprisingly, the solvent methanol is by far superior to water even though the solubility of Lac is poor in methanol. We conclude that the solubility of a saccharide is not a determining factor for the amination according to Kochetkov and Likhoshertov. As well as for GalNAc, we obtained the highest yield for Lac (83.6%) at the highest temperature (60 °C) and the lowest concentration (10 mg/mL) with ammonium carbonate and methanol. Calculated optimized conditions for Lac are a temperature of 60 °C and a concentration of 58 mg/mL with ammonium carbonate and methanol. After conducting the optimized experiment, we could indeed increase the yield to 91.1%. The deviation from the predicted yield of 100.4% lies within the experimental error. The prediction lies above 100% as solely the target was set to 100% and not the maximum (the maximum cannot equal the target in MODDE). Overall, the DoE approach successfully improved the yield of aminated Lac.

**Figure 4:**
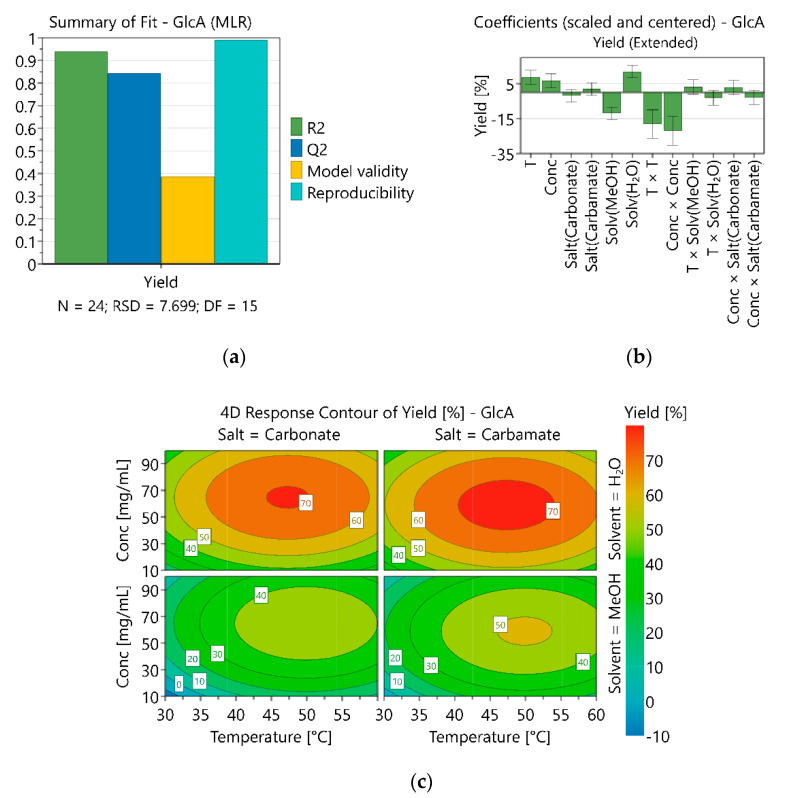
Plots of the model for GlcA generated by MODDE: (**a**) Summary of fit represents a good model; (**b**) plot of coefficient values for scaled and centered factors show significant factors according to the model; (**c**) the 4D response contour plot of yield predicts yields of amination in dependence on qualitative and quantitative factors.

GlcA is a uronic acid and therefore our model compound for this class of saccharides. After amination a zwitter-ionic compound is produced. In humans, GlcA is mostly found in glucosaminoglycans. The summary of fits for GlcA displays excellent values of R^2^ = 0.94 and Q^2^ = 0.84 (Figure 4a). In comparison, the model validity is rather low (0.39) which may be due to the high reproducibility value of 0.99 and not due to a real lack of fit. Significant terms for GlcA are temperature, concentration, both aminating agents ammonium carbonate and ammonium carbamate, the solvents methanol and water, the quadratic term of temperature × temperature, concentration × concentration, temperature × methanol, temperature × water, concentration × ammonium carbonate and concentration × ammonium carbamate (Figure 4b). The amination of GlcA seems strongly dependent on temperature, concentration and choice of solvent. Interestingly, for GlcA further factors are significant including the nature of ammonium salt and its dependency on the concentration. From the 4D contour plot (Figure 4c), it is evident that water works better than methanol for the amination of GlcA. Regarding the aminating agent, ammonium carbamate appears to be the preferred choice. In experiments, the highest yield (81.6%) was achieved at 45 °C, 55 mg/mL with ammonium carbamate in water. Optimized reactions conditions are 47 °C, 59 mg/mL, ammonium carbamate and water with a predicted yield of 73.8%. The optimized experimental conditions resulted in a yield of 60.3%. The predicted yield is lower than the highest yield found in previous experiments and, furthermore, does not correlate to the yield found. This hints at statistical problems of the model even though the prediction value Q^2^ was very good. Moreover, in this model yields above 73.8% are not achievable although Ghadban et al. did attain yields of up to 89% [56]. We suggest a larger set of experiments and a wider range of reaction parameters for the reaction conditions with water and ammonium carbamate to improve the model.

**Figure 5:**
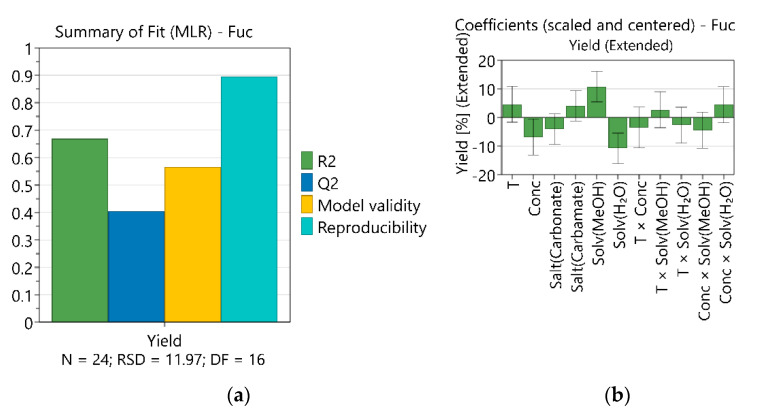

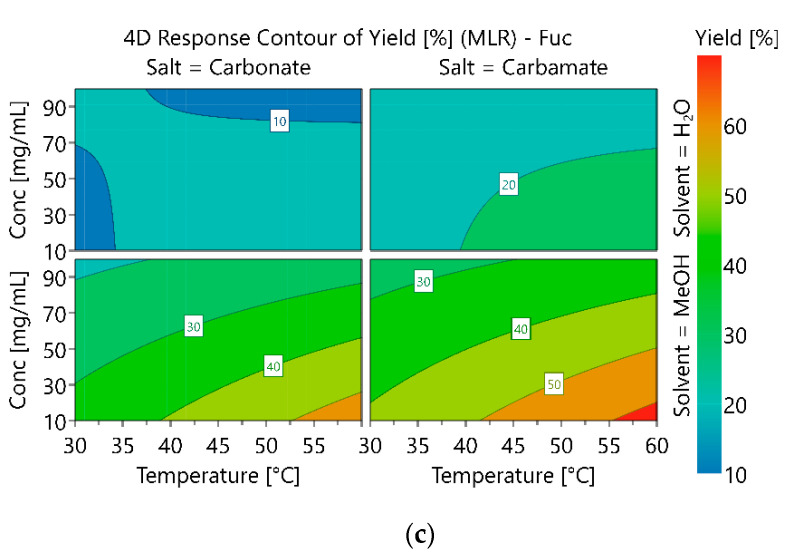
Plots of the model for Fuc generated by MODDE: (**a**) Summary of fit shows a lower significance of the model; (**b**) plot of coefficient values for scaled and centered factors show significant factors according to the model; (**c**) the 4D response contour plot of yield predicts yields of amination in dependence on qualitative and quantitative factors.

Our model compound for desoxy-sugars is 6-desoxy galactose, better known as Fuc. Fuc-based derivatives could, for example, be important for inhibiting the formation of *Pseudomonas aeruginosa* biofilms. Additionally, it is a very abundant sugar in human milk oligosaccharides. The summary of fits for Fuc presents a good R^2^ value of 0.67 and a Q^2^ value of 0.40 (Figure 5a). The model validity is 0.57 and the reproducibility has a high value of 0.90. Thus, this is a model of lower significance. Although the histogram of the Fuc experiments exhibits positive skewness (Supplementary Materials), no transformation was performed as the model for Fuc produced better values than without transformation. MODDE displays the significant terms temperature, concentration, both salts ammonium carbonate and carbamate, the solvents methanol and water, the square term of temperature × concentration, temperature × methanol, temperature × water, concentration × methanol and concentration × water (Figure 5b). Thus, the amination of Fuc greatly depends on temperature, concentration and nature of solvent. Furthermore, the choice of ammonium salt and the influence of temperature and concentration on the solvents affect the yield, too. In the 4D contour plot of yield (Figure 5c), when comparing the solvents, we see that overall methanol leads to higher yields. Water seems to work poorly for the amination of Fuc. Regarding the aminating agent, the highest yield is obtained with ammonium carbamate. Yield increases with rising temperature and decreasing concentration. Hence, a further increase of the temperature and decrease of the concentration might improve the yield. We obtained the highest yield of 69.8% at 60 °C and 10 mg/mL with ammonium carbamate and methanol. Optimized amination conditions for Fuc are the exact reaction conditions with a predicted yield of 63.4%. The predicted yield is lower than the already obtained yield but lies within the experimental error. This still indicates a flawed model, which correlates to the rather low prediction value Q^2^. However, the optimized reaction conditions coincide with the performed conditions with the best result. To further optimize the amination of Fuc, the model should be improved by producing more data of experiments where methanol is used as the solvent.

Overall, for the amination of carbohydrates according to the Kochetkov and Likhoshertov method, the reaction temperature has the most significant influence on the yield. The contour plots of MODDE indicate that higher yields are achievable at temperatures above 60 °C. In contrast, according to Bejugam et al. higher temperatures generally lead to an increased formation of side products and, therefore, lower yields [43]. Side products such as dimers were not analyzed but as such would usually not disturb subsequent reactions like, for example, (meth-)acrylations [37,38]. Though colorations of yellow and reddish brown were observed after reaction at temperatures above 50 °C, this did not seem to diminish the yield of glycosylamines. Concentration of saccharide does affect the yield but not strongly. Surprisingly, suspension of highly concentrated reactions did not necessarily decrease the yield even though thorough stirring was not always possible; microwave irradiation and excess amount of ammonium salt was enough to aminate the saccharides in the suspension. Choice of solvent also influences the yield and depends on the nature of saccharide. Contrary to our expectation, methanol seems to be the superior solvent for amination of saccharides except for GlcA. The poor solubility of saccharides and aminating agents in methanol shows no negative influence on the yield. In conclusion, the solubility of starting material does not seem to affect the amination. A possible explanation is that the temperature and microwave irradiation are enough to dissolve, aminate, or both, the saccharides in methanol. Moreover, water can lead to hydrolysis and hence decrease the actual yield during purification or analysis. Regarding the first-time use of microwave assisted amination according to Likhoshertov, good yields of up to 81.6% could be obtained within 90 min as opposed to the 4–48 h from the traditional procedure [40]. Thus, microwave irradiation allows a great reduction of reaction time for the amination according to Likhoshertov, too. Generally, the nature of aminating agent can have an influence depending on selected saccharide, solvent, or both. This shows that both microwave-assisted syntheses work equally well as amination reaction for oligosaccharides and is not surprising since both ammonium salts are volatile and generate ammonia. Furthermore, we repeated experiment Am-I-01 (Table 1) with a 33-fold batch size in a 1 L PTFE vessel as its reaction conditions lead to the highest yield achieved. In this way we investigated the scalability of the process in principle. The initial yield of 64% dropped significantly even if the reaction time was doubled. No amine was found in NMR spectrum and only little amine was found by TLC. This may be due to different distribution of microwave irradiation in the larger volume, which could be another parameter for future investigations. However, we can also conclude that alterations of microwave distribution can be one of the reasons why yields from different publications and our yields may differ.

The DoE approach enabled a reduced number of experiments; however, if the model is insufficient, more experiments have to be conducted to improve the model. Predictions of the software support the direction of future experiments, namely, which solvent or aminating agent to use. We suggest additional experiments with higher reaction temperatures to further optimize the amination of saccharides. We consider investigating the reaction time to be worthwhile as well.

## 3. Materials and Methods

### 3.1. Materials

All chemicals were purchased from commercial sources. Water was double deionized by a Milli-Q purification system (18.2 MΩ·cm, Millipore Quantum TEX, Darmstadt, Germany). *N*-Acetyl-d-galactosamine (GalNAc; ≥99%, Carbosynth, Comptun, UK), d-lactose monohydrate (Lac; ≥96%, Carbosynth), d-glucuronic acid (GlcA; ≥98%, Carbosynth), l-(−)-fucose (Fuc; ≥98%, Carbosynth), ammonium carbamate (H_2_NCOONH_4_; 99%, Aldrich, Steinheim, Germany), ammonium carbonate ((NH_4_)_2_CO_3_; ≥30.5% NH_3_, extra pure, Carl Roth, Karlsruhe, Germany), methanol (MeOH; ≥98.8%, VWR, Darmstadt, Germany), deuterium oxide (D_2_O; 99.9%, Deutero, Kastellaun, Germany) were used as received.

### 3.2. Methods

#### 3.2.1. Design of Experiments (DoE)

The software MODDE version 12.1 (Sartorius Stedim Data Analytics AB, Malmö, Sweden) for generation and evaluation of statistical experimental designs was used to optimize synthesis conditions. We selected concentration of saccharide (Conc) and reaction temperature (T) as quantitative factors. The aminating agents (Salt) and solvents (Solv) represented our qualitative factors. We investigated the yield of the respective glycosylamine as response and set 100% yield as target. We chose the D-optimal design (with highest G-efficiency) and quadratic model to generate a set of experiments for optimization. This set includes two replicates for testing reproducibility. The models were fitted with multiple linear regression (MLR) analysis.

#### 3.2.2. Nuclear Magnetic Resonance (NMR)

^1^H-NMR spectra were recorded on a Bruker Neo Avance 400 MHz spectrometer (Bruker, Ettlingen, Germany) to identify the glycosylamines and determine their yields. We measured all spectra in D_2_O. Yields of the respective glycosylamines were determined by evaluating the ratio between the integral of proton signals, that both starting material and glycosylamine share, and the integral that is solely specific to the respective glycosylamine. In case of GalNAcNH_2_, we examined the ratio between the integral of the methyl group proton signal of GalNAc/GalNAcNH_2_ (H-7; 3 H) and the integral of the anomeric proton signal of the GalNAcNH_2_ (n H; yield of glycosylamine = n × 100%). For LacNH_2_, the ratio between the integral of the proton peak H-7 (1 H) and the integral of the anomeric proton signal of LacNH_2_ (n H). As peaks of the anomeric proton of GlcA and its amination product overlap, we performed global spectral deconvolution (GSD) for analysis. The integral of the peaks of the protons H-2 to H-5 (4 H) were compared with the integral of the anomeric proton signal of GlcANH_2_ (n H). The yield of FucNH_2_ was determined by analyzing the ratio between the integral of the methyl group proton signal H-6 (3 H) and the integral of the anomeric proton peak of FucNH_2_ (n H).

#### 3.2.3. Electrospray Ionization Mass Spectrometry (ESI-MS)

ESI-MS spectra were recorded on a PerkinElmer Flexar SQ 300 MS (Rodgau, Germany). We dissolved samples in acetonitrile/water mixture (50:50) with 0.1% formic acid. The measurements were performed at 300 °C with a flow rate of 15 μL min^−1^.

#### 3.2.4. Synthesis of Glycosylamines

Amination of saccharides were performed in a START 1500 rotaPREP microwave reactor (MLS GmbH, Leutrich, Germany). The respective saccharide is charged in a 50 mL-glass vessel and stirred with solvent. Afterwards, the ammonium salt is added under stirring and the reaction vessel is transferred to the microwave reactor. We set the reaction time to 90 min. The heating phase to our desired reaction temperature was set to 5 min. Volume of solvent was constantly 8 mL to ensure equal distribution of microwave irradiation for every experiment. We varied reaction temperature, concentration of saccharide, solvent and aminating agent according to Table 1. The last experiment is repeated three times in total for testing reproducibility. After reaction, samples prepared in MeOH were first concentrated by rotary evaporation at 40 °C and 300 mbar, followed by complete drying under high vacuum over several days or until most of the ammonium salt is removed. Aqueous reaction mixtures were lyophilized after reaction for several days or until most of the ammonium salt is removed. We yielded (hygroscopic) β-glycosylamines and stored them in nitrogen atmosphere at 4 °C.

The numbering of experiments starts with “Am” for amination, followed by the designated roman numeral of saccharide, GalNAc (I), Lac (II), GlcA (III)and Fuc (IV), and ends with the number of experiment. For example, Am-IV-03 refers to the amination of Fuc with the reaction conditions of experiment number 03. Experiments with optimized reaction conditions generated by MODDE carry the experiment number 0 (Table 2).

## 4. Conclusions

We optimized amination conditions for *N*-acetyl-d-galactosamine, d-lactose, d-glucuronic acid and l-(−)-fucose using DoE approach. Additionally, we showed that the acceleration of the amination according to Likhoshertov is possible by microwave irradiation. It is very apparent that optimized reaction conditions for one saccharide do not apply in the same way for other saccharides. Due to the relatively small number of experiments most models were lacking to some extent. However, the DoE approach supported the direction of which reaction parameters are worth further testing, including their quantitative and qualitative ranges or properties, respectively. The model for the amination of Lac provided a great improvement of yield. We observed strong indication that high temperatures are preferable for the amination. For future experiments, we suggest additional data of experiments with our found, most beneficial conditions to improve the models, testing of reaction time and of elevated temperatures.

## Figures and Tables

**Table 1:** Reaction conditions and yields of amination. Highest yields are indicated by underscores.

Exp No	T (°C)	(mg/mL)	Salt	Solvent	Yield (%)			
					Am-I GalNAcNH_2_	Am-II LacNH_2_	Am-III GlcANH_2_	Am-IV FucNH_2_
01	60	10	(NH_4_)_2_CO_3_	MeOH	64.2	83.6	7	60.5
02	30	100	(NH_4_)_2_CO_3_	MeOH	53.7	33	0.9	12.4
03	60	100	(NH_4_)_2_CO_3_	MeOH	42.2	68	33.6	21.8
04	30	40	(NH_4_)_2_CO_3_	MeOH	43.1	46.4	2.1	45
05	40	10	(NH_4_)_2_CO_3_	MeOH	30.9	20.8	1.6	25
06	30	10	H_2_NCOONH_4_	MeOH	33.6	11.8	3.3	42.6
07	60	10	H_2_NCOONH_4_	MeOH	51.6	81.4	12	69.8
08	30	100	H_2_NCOONH_4_	MeOH	44.9	27.4	3	32.4
09	60	100	H_2_NCOONH_4_	MeOH	41.9	79.2	23.6	38.8
10	45	55	H_2_NCOONH_4_	MeOH	57.4	79.7	53.1	26
11	30	10	(NH_4_)_2_CO_3_	H_2_O	39.1	16.7	16.8	16.2
12	60	10	(NH_4_)_2_CO_3_	H_2_O	27.3	26.2	35.7	18.2
13	30	100	(NH_4_)_2_CO_3_	H_2_O	26.5	11.5	37.3	9
14	60	70	(NH_4_)_2_CO_3_	H_2_O	37.8	42.4	54.6	10.3
15	50	100	(NH_4_)_2_CO_3_	H_2_O	20.4	44.3	51.9	8.4
16	30	10	H_2_NCOONH_4_	H_2_O	41.2	8.8	18.3	6.9
17	60	100	H_2_NCOONH_4_	H_2_O	50.5	30.2	46.8	12.4
18	30	70	H_2_NCOONH_4_	H_2_O	29.4	13.5	47.8	8.7
19	60	40	H_2_NCOONH_4_	H_2_O	44.2	21.5	44.4	17.1
20	50	10	H_2_NCOONH_4_	H_2_O	30	20.1	46.1	11.1
21	40	100	H_2_NCOONH_4_	H_2_O	34.4	24.7	40.3	8.7
22a	45	55	H_2_NCOONH_4_	H_2_O	17	32.5	77.7	33.3
22b	45	55	H_2_NCOONH_4_	H_2_O	20.4	74.1	77	41.8
22c	45	55	H_2_NCOONH_4_	H_2_O	18	62.5	81.6	31.2

**Table 2:** Optimized reaction conditions and yields generated by MODDE.

Exp No	T (°C)	(mg/mL)	Salt	Solvent	Predicted Yield (%)	Found Yield (%)
Am-I-0/-01	60	10	(NH_4_)_2_CO_3_	MeOH	54.7	64.2
Am-II-0	60	58	(NH_4_)_2_CO_3_	MeOH	100.4	91.1
Am-III-0	47	59	H_2_NCOONH_4_	H_2_O	73.8	60.3
Am-IV-0/-07	60	50	H_2_NCOONH_4_	MeOH	63.4	69.8

## Supplementary Materials

The following are available online, Figure S1. Overview plot of yields of GalNAcNH_2_. Replicates are indicated in blue, Figure S2. Histogram of yields of GalNAcNH_2_. Skewness test not triggered, Figure S3. Plot of GalNAcNH_2_ with residuals of yields versus the normal probability of the distribution, Figure S4. Plot of observed values versus predicted values for yields of GalNAcNH_2_, Figure S5. Overview plot of yields of LacNH_2_. Replicates are indicated in blue, Figure S6. Histogram of yields of LacNH_2_. Skewness test not triggered, Figure S7. Plot of LacNH_2_ with residuals of yields versus the normal probability of the distribution, Figure S8. Plot of observed values versus predicted values for yields of LacNH_2_, Figure S9. Overview plot of yields of GlcANH_2_. Replicates are indicated in blue, Figure S10. Histogram of yields of GlcANH_2_. Skewness test not triggered, Figure S11. Plot of GlcANH_2_ with residuals of yields versus the normal probability of the distribution, Figure S12. Plot of observed values versus predicted values for yields of GlcANH_2_, Figure S13. Overview plot of yields of FucNH_2_. Replicates are indicated in blue, Figure S14. Histogram of yields of FucNH_2_. Skewness test triggered. No transformation performed, Figure S15. Plot of FucNH_2_ with residuals of yields versus the normal probability of the distribution, Figure S16. Plot of observed values versus predicted values for yields of FucNH_2_.
