# Supplementary material for: Optimization of the Microwave Assisted Glycosylamines Synthesis Based on a Statistical Design of Experiments Approach
Source: Molecules. 2020 Nov 4;25(21):5121. doi: 10.3390/molecules25215121 (PMC7663175; doi:10.3390/molecules25215121)
Supplement: Supplementary file 1 [file molecules-25-05121-s001.pdf]

Supporting information

Optimization of the Microwave Assisted Glycosylamines Synthesis Based on a Statistical Design of Experiments Approach

Jo Sing Julia Tang [1], Kristin Schade [1], Lucas Tepper [2], Sany Chea [1], Gregor Ziegler [1] and Ruben R. Rosencrantz [1,*]

[1] Fraunhofer Institute for Applied Polymer Research IAP, Biofunctionalized Materials and (Glyco)Biotechnology, Geiselbergstr. 69, 14476 Potsdam, Germany
[2] Freie Universität Berlin, Department of Physics, Arnimallee 14, 14195 Berlin, Germany

* Correspondence: ruben.rosencrantz@iap.fraunhofer.de; Tel.: +49-331-568-3203

1-Amino-1-deoxy-β-D-N-acetylgalactoside (**Am-I-01**). ^{1}H-NMR (D_2O, 400 MHz): δ 5.21 (d, *J* = 3.6 Hz, 0.04H α-H1 (starting material)), 4.60 (d, *J* = 7.6 Hz, 0.05 H, β-H1 (starting material)), 4.30-3.51 (m, 6 H), 4.06 (d, *J* = 9.2 Hz, 0.64 β-H1), 2.03 (s, 3 H); ESI MS, calcd. for $C_8H_{16}N_2O_5$: $[M + H]^+$ 221.11, found 221.45 $[M + H]^+$.

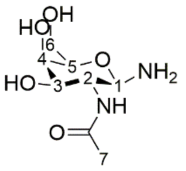

Am-I

1-Amino-1-deoxy-β-D-lactoside (**Am-II-01**). ^{1}H-NMR (D_2O, 400 MHz): δ 5.21 (d, *J* = 3.6 Hz, 0.05 H, α-H1 (starting material)), 4.65 (d, *J* = 8.0 Hz, 0.06 H, β-H1 (starting material)), 4.43 (d, *J* = 7.8 Hz, 1 H, β-H7), 4.10 (d, *J* = 8.8 Hz, 0.84 H, β-H1), 3.97-3.48 (m, 11 H), 3.19 (m, 0.82 H); ESI MS, calcd. for $C_{12}H_{24}NO_{10}$: $[M + H]^+$ 342.14, found 342.46 $[M + H]^+$.

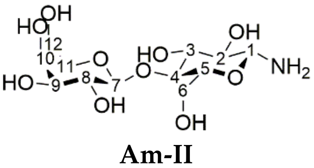

Am-II

1-Amino-1-deoxy-β-D-glucopyranuronoside (**Am-III**). ^{1}H-NMR (D_2O, 400 MHz): δ 5.24 (d, *J* = 3.7 Hz,4 H, α-H1 (starting material)), 4.64 (d, *J* = 7.9 Hz, β-H1 (starting material), 4.09 (d, *J* = 8.8 Hz, 0.82 H, β-H1), 3.12-4.34 (m, 4 H); ESI MS, calcd. for $C_6H_{12}NO_6$: $[M + H]^+$ 194.07, found 194.43 $[M + H]^+$.

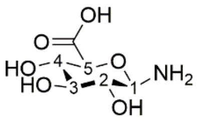

Am-III

1-Amino-1-deoxy-β-L-fucose (**Am-IV**). ^{1}H-NMR (D_2O, 400 MHz): δ 4.15-4.03 (m, 0.26 H), 3.99 (d, *J* = 8.8 Hz, 0.70 H, β-H1), 3.36-3.94 (m, 3.20 H), 3.32 (t, *J* = 8.8 Hz, 0.58 H) 1.20 (d, *J* = 6.4 Hz, 3 H); ESI MS, calcd. for $C_6H_{14}NO_4$: $[M + H]^+$ 164.09, found 164.38 $[M + H]^+$.

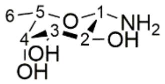

Am-IV

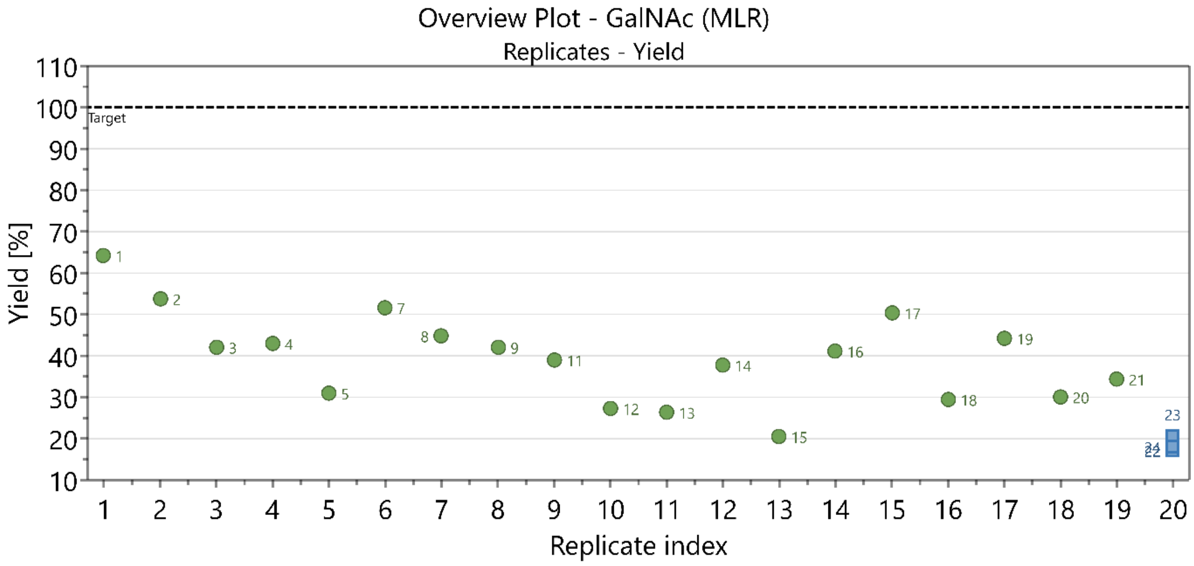

Figure S1. Overview plot of yields of GalNAcNH2. Replicates are indicated in blue.

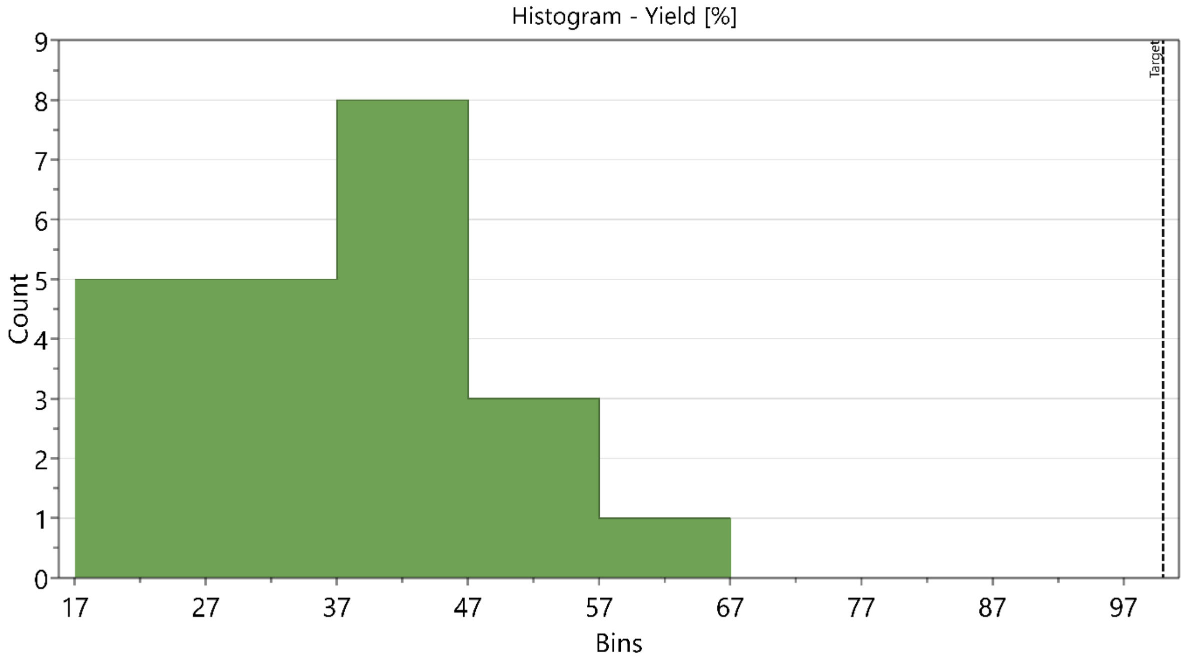

N=22; DF=14; Cond. no.=5.862; RSD=6.884; Q2=0.498

Figure S2. Histogram of yields of GalNAcNH2. Skewness test not triggered.

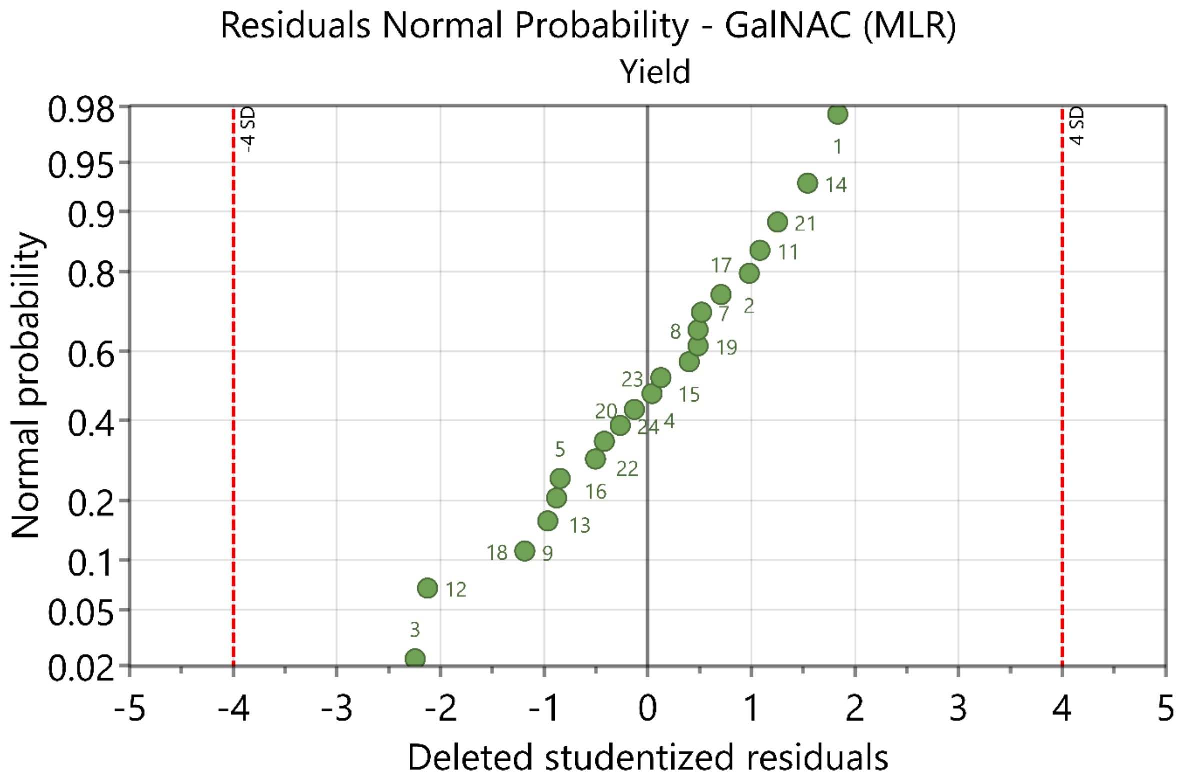

Figure S3. Plot of GalNAcNH2 with residuals of yields versus the normal probability of the distribution.

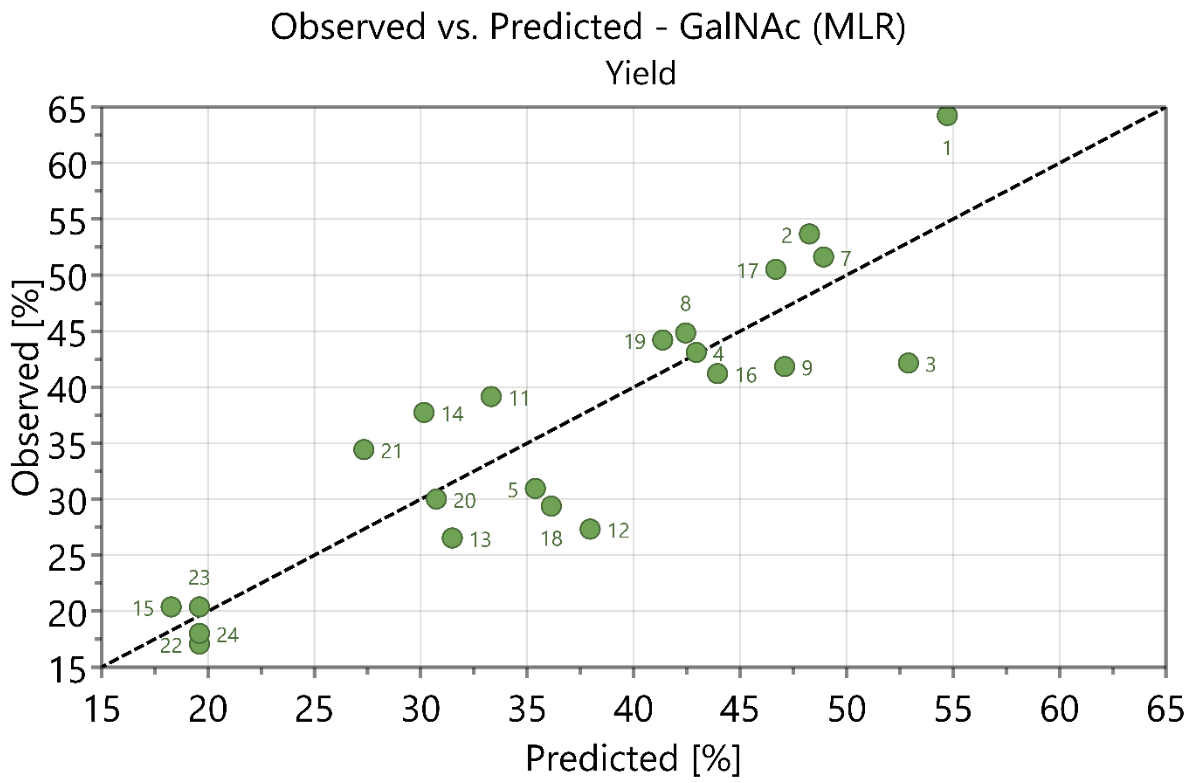

Figure S4. Plot of observed values versus predicted values for yields of GalNAcNH2.

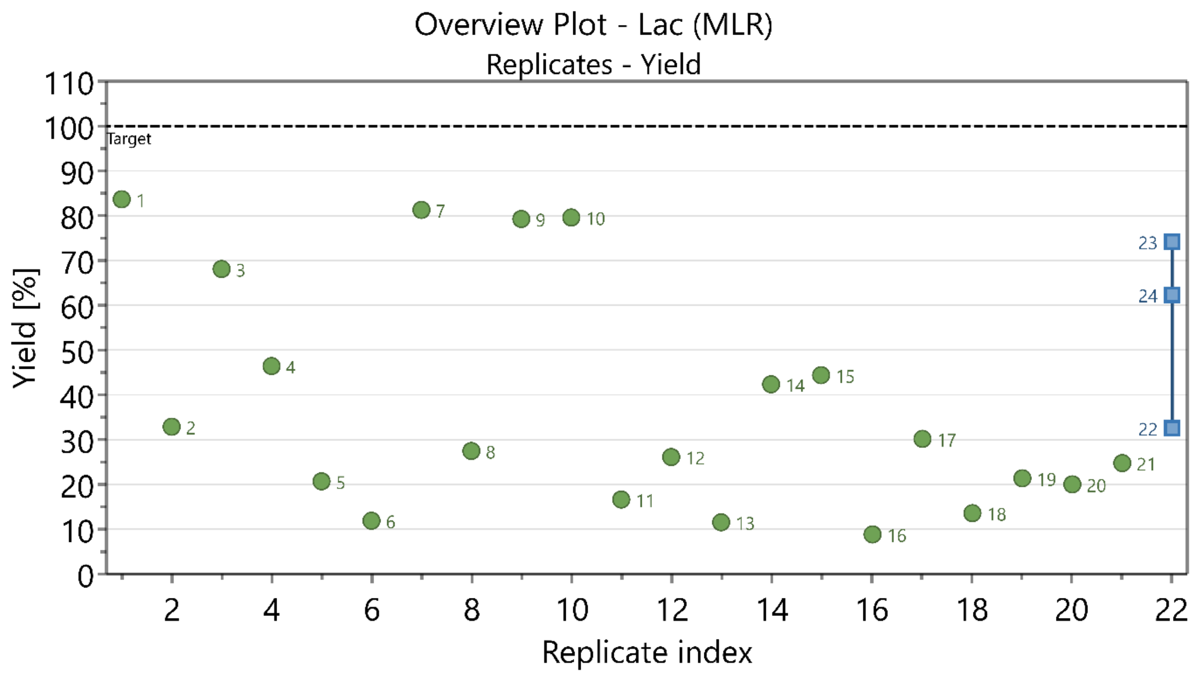

Figure S5. Overview plot of yields of LacNH2. Replicates are indicated in blue.

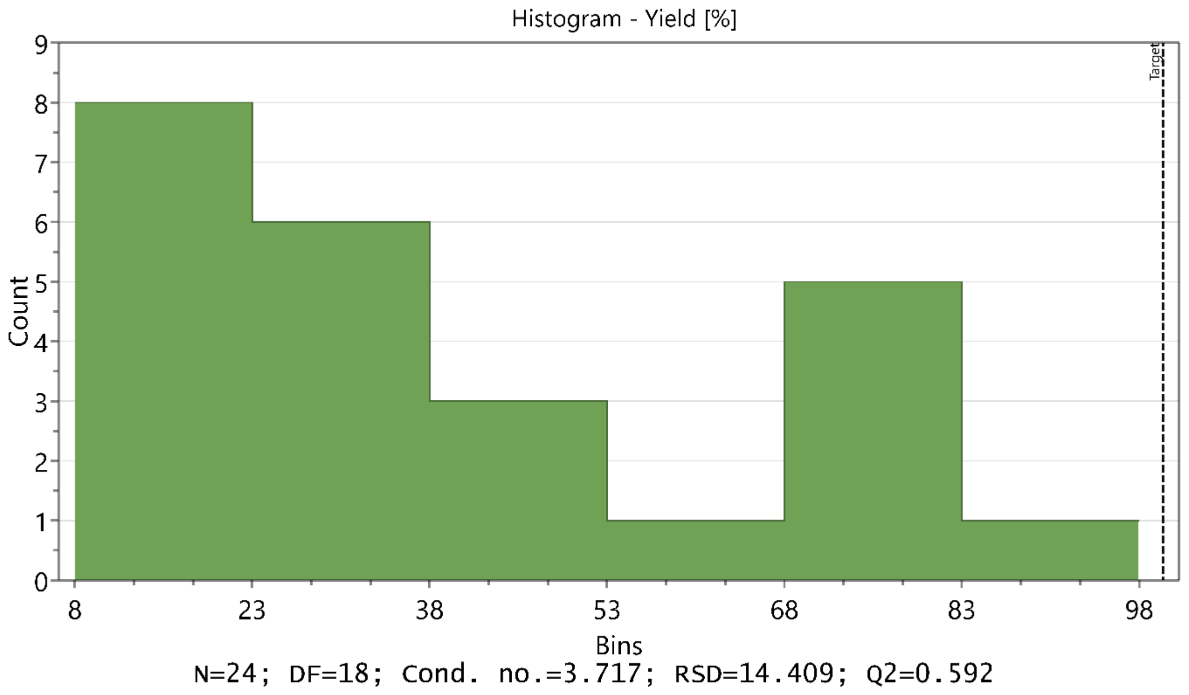

Figure S6. Histogram of yields of LacNH2. Skewness test not triggered.

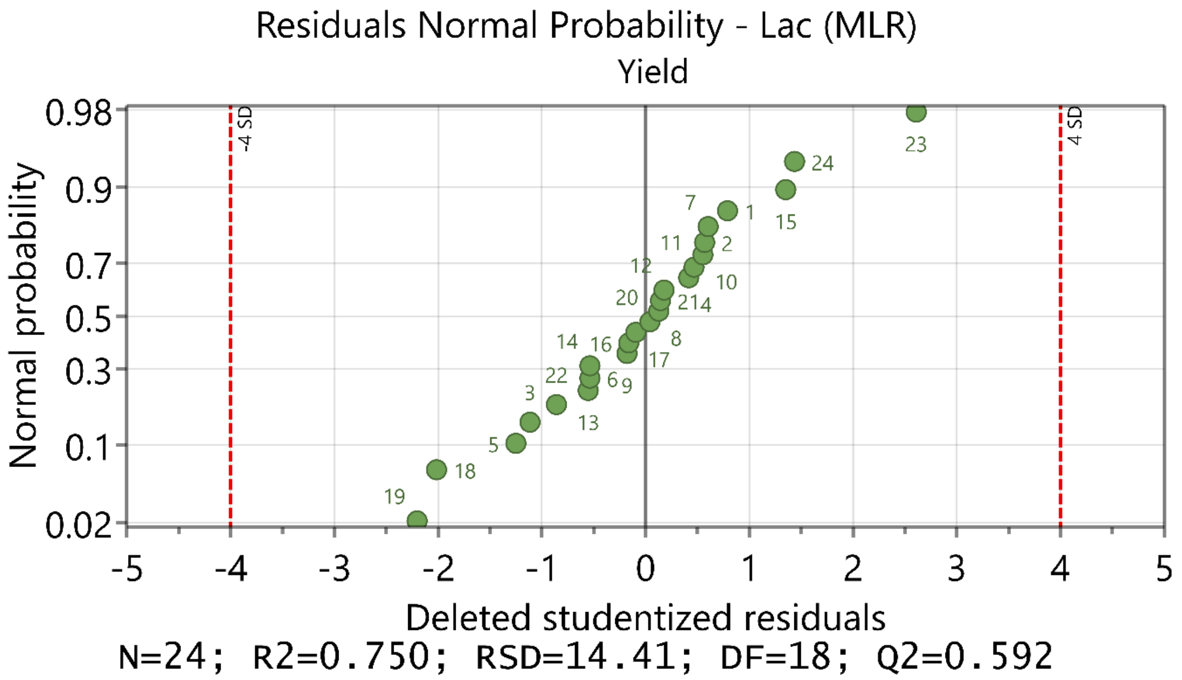

Figure S7. Plot of $LacNH_2$ with residuals of yields versus the normal probability of the distribution.

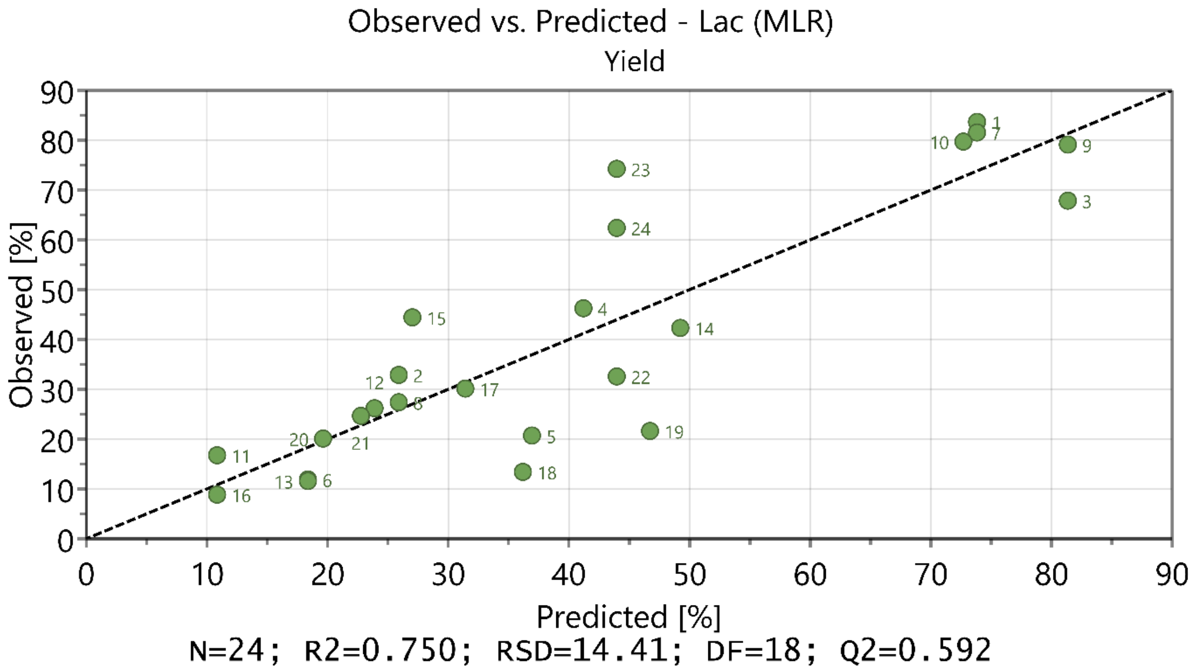

Figure S8. Plot of observed values versus predicted values for yields of $LacNH_2$.

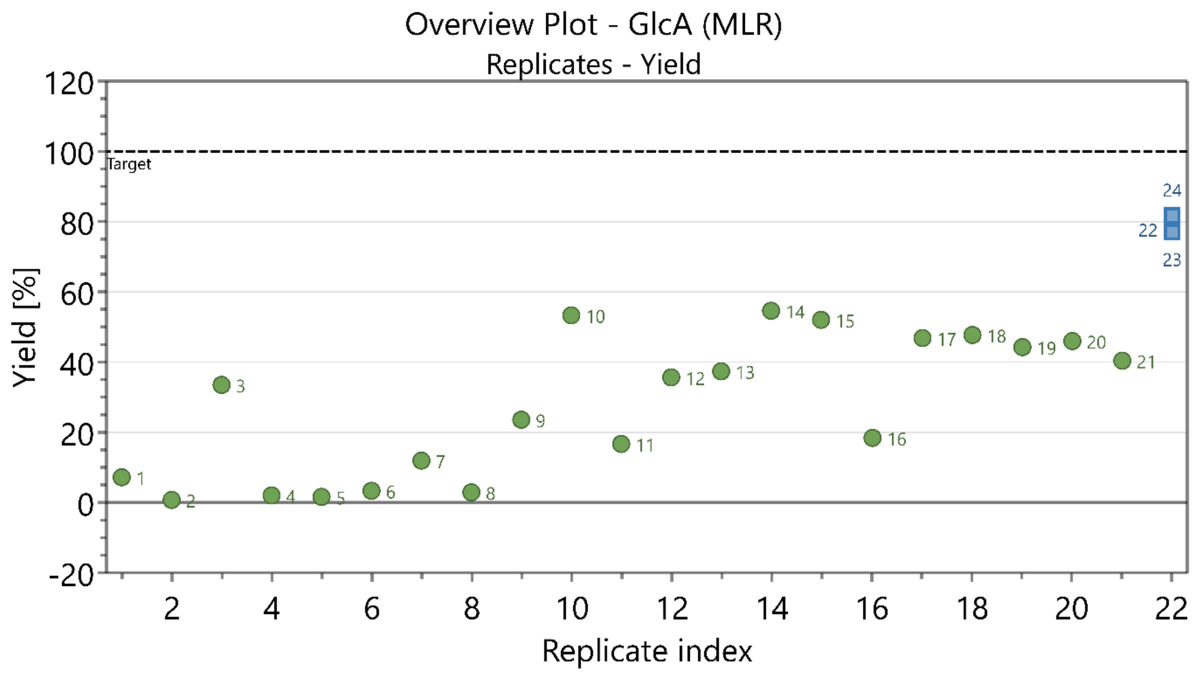

Figure S9. Overview plot of yields of $GlcANH_2$. Replicates are indicated in blue.

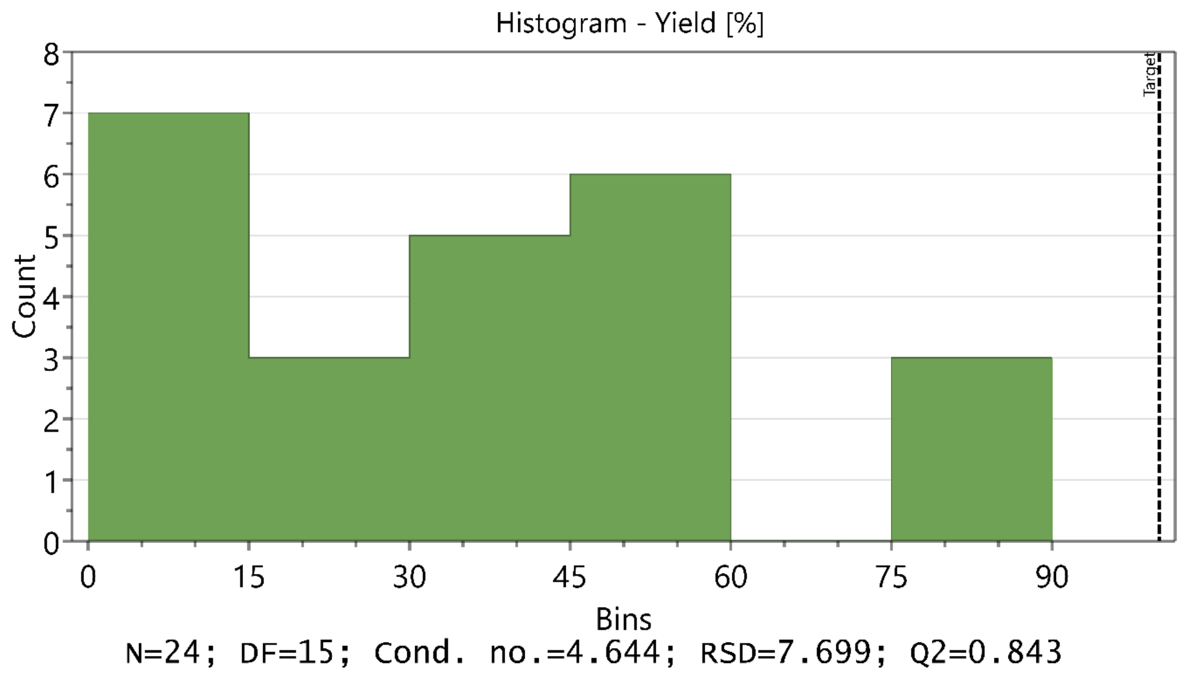

Figure S10. Histogram of yields of $GlcANH_2$. Skewness test not triggered.

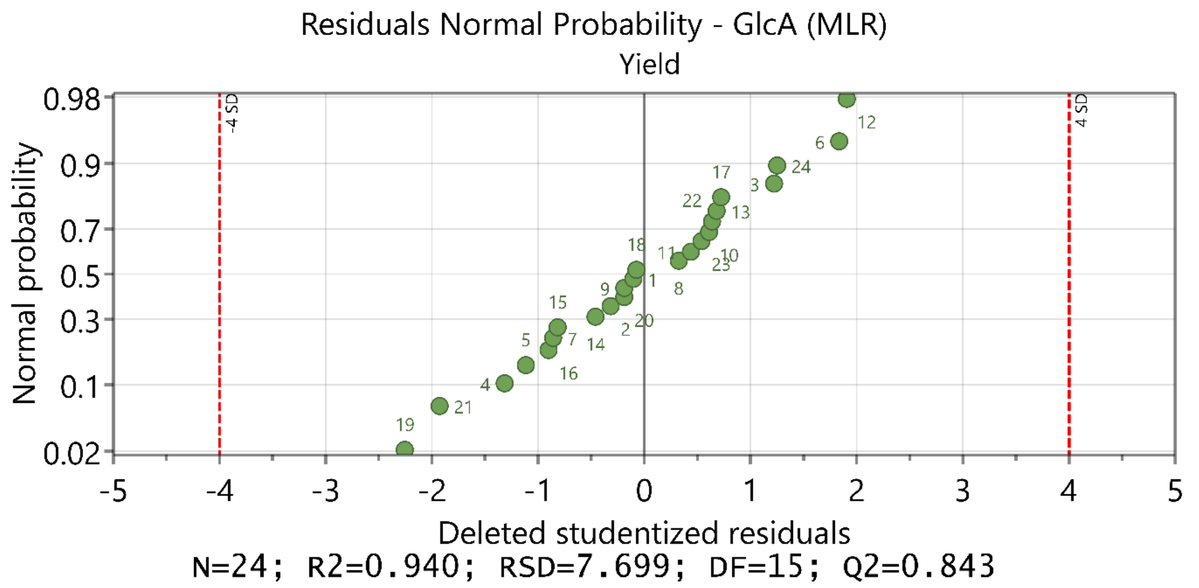

Figure S11. Plot of GlcANH2 with residuals of yields versus the normal probability of the distribution.

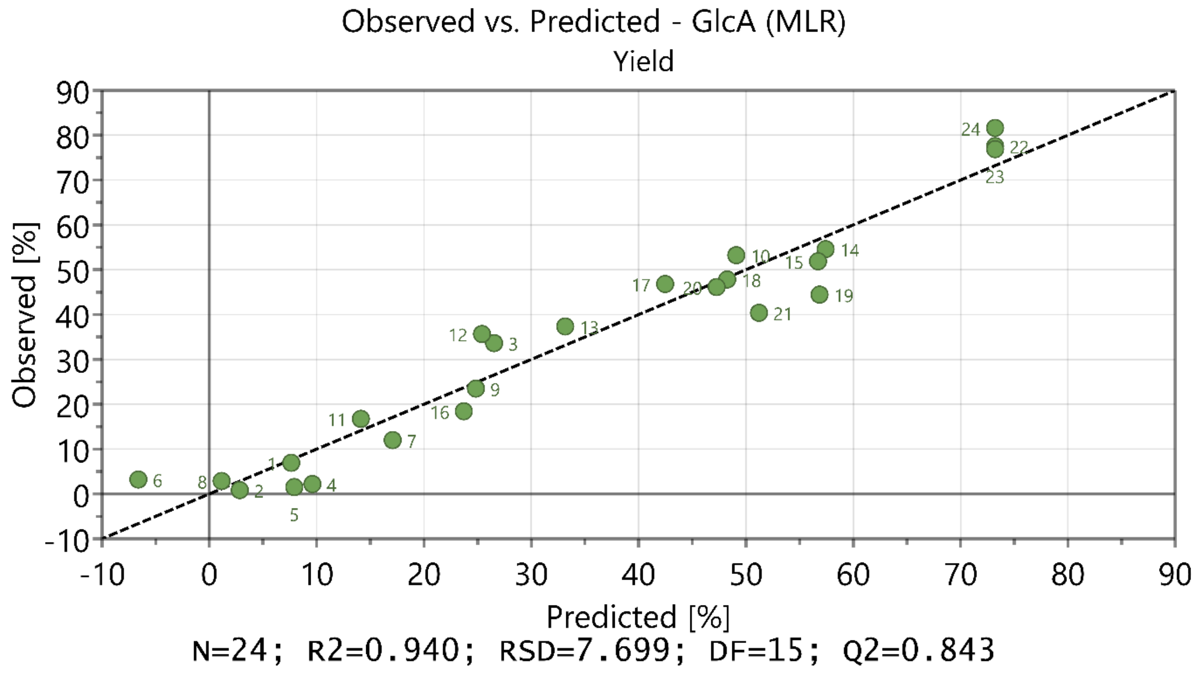

Figure S12. Plot of observed values versus predicted values for yields of GlcANH2.

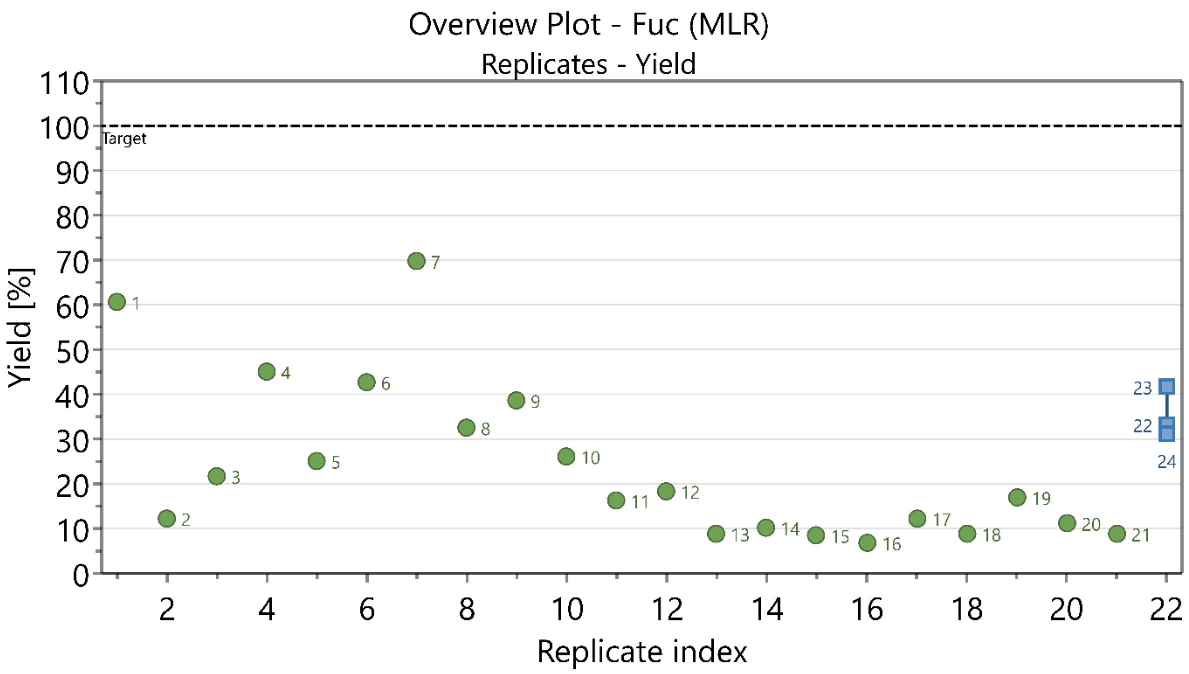

Figure S13. Overview plot of yields of $FucNH_2$. Replicates are indicated in blue.

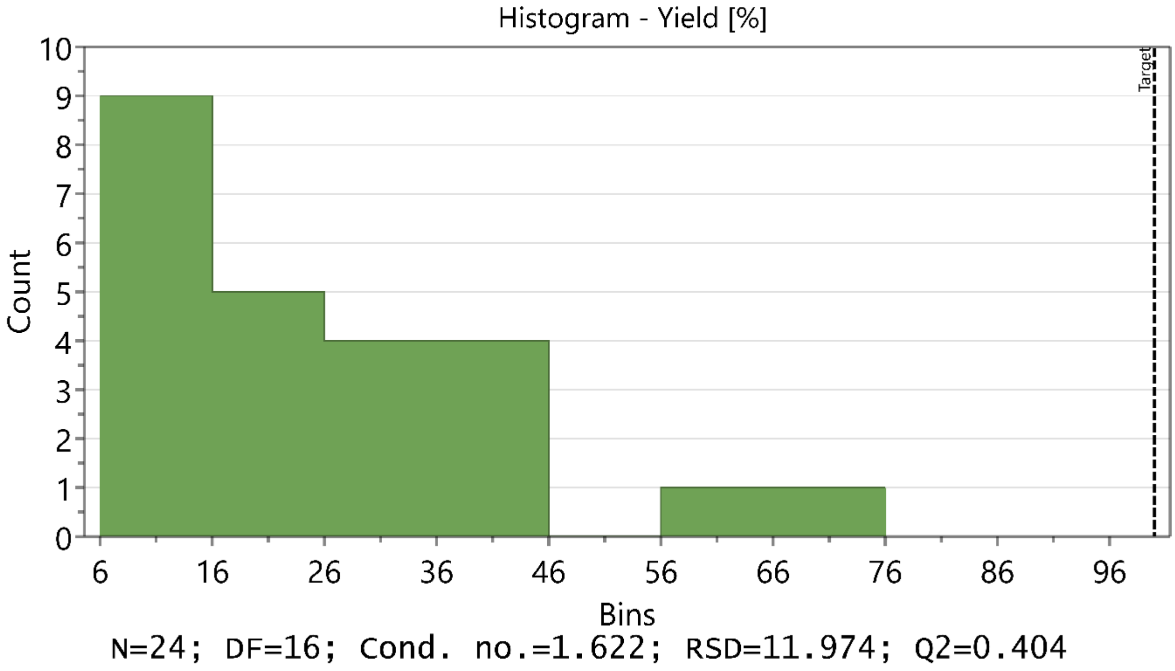

Figure S14. Histogram of yields of $FucNH_2$. Skewness test triggered. No transformation performed.

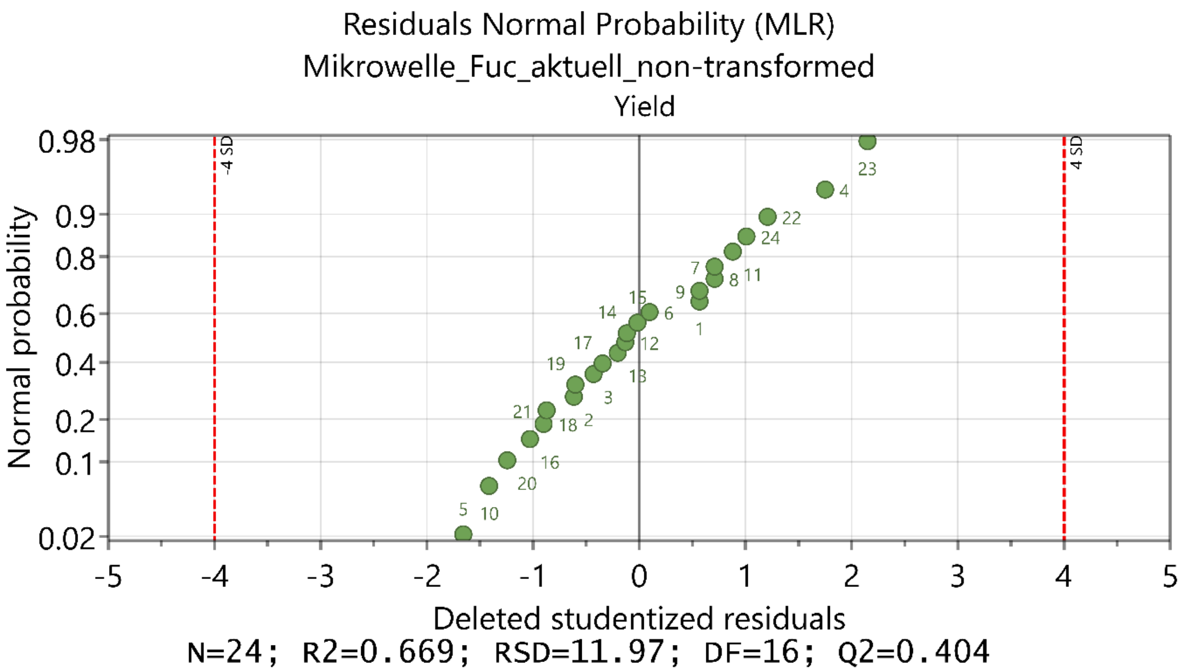

Figure S15. Plot of $FucNH_2$ with residuals of yields versus the normal probability of the distribution.

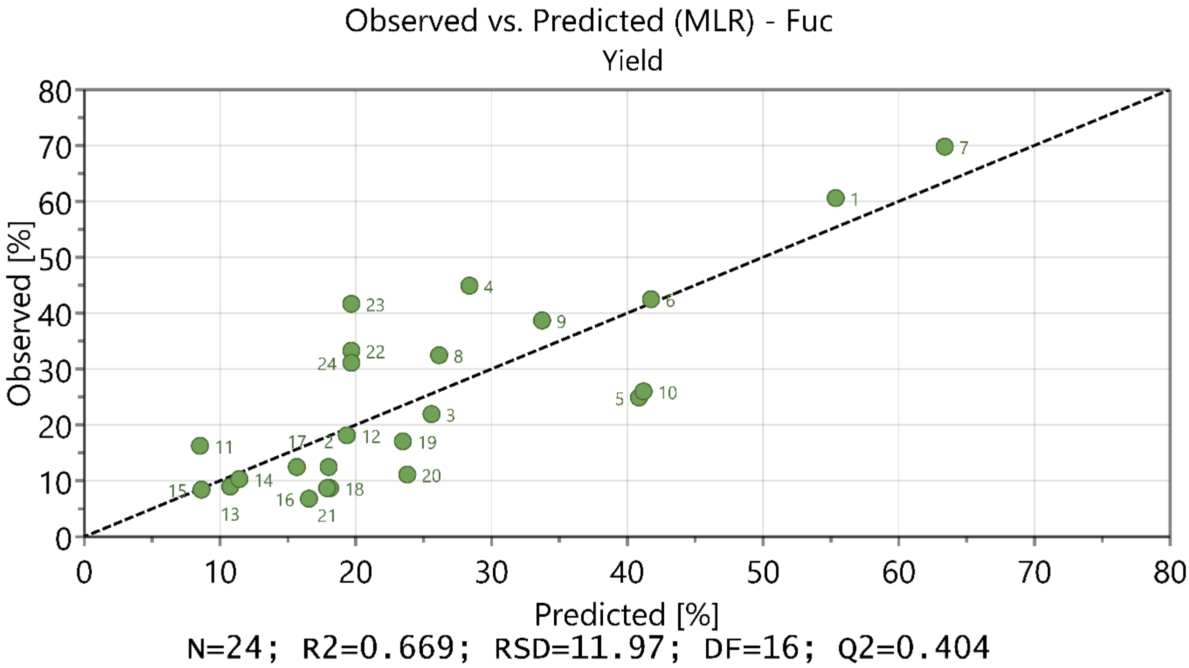

Figure S16. Plot of observed values versus predicted values for yields of $FucNH_2$.
